# Radiographic damage in hands and wrists of patients with juvenile idiopathic arthritis after 29 years of disease duration

**DOI:** 10.1186/s12969-017-0151-7

**Published:** 2017-04-11

**Authors:** Anne M. Selvaag, Eva Kirkhus, Lena Törnqvist, Vibke Lilleby, Hanne A. Aulie, Berit Flatø

**Affiliations:** 1 Department of Rheumatology, Oslo University Hospital, Rikshospitalet, Post-box 4950, Nydalen, 0424 Oslo, Norway; 2 Department of Radiology and Nuclear Medicine, Oslo University Hospital, Rikshospitalet, Oslo, Norway; 3 Institute of Clinical Medicine, Faculty of Medicine, University of Oslo, Oslo, Norway; 4 Present address: Department of Radiology, Linköping University, Linköping, Sweden; 5 Present address: Department of Internal Medicine, Diakonhjemmet Hospital, Oslo, Norway

**Keywords:** Radiography, Juvenile idiopathic arthritis, Follow-up studies, Disease progression, Prognosis, Risk factors, Longitudinal studies

## Abstract

**Background:**

There are few studies on radiographic outcome after long-term disease duration in juvenile idiopathic arthritis (JIA). We wanted to evaluate 29-year radiographic outcome in hands/wrists and predictors of damage in patients with long-term active JIA.

**Methods:**

Patients diagnosed from 1980 to 1985, who had active disease at 15-, 23- or 29-year follow-up and arthritis in the wrists during the disease course, were reexamined with radiographs of hands/wrists. We used the adapted version of the Sharp van der Heijde (aSvdH) score and Carpal Height Ratio (CHR) to evaluate radiographic outcome.

**Results:**

Sixty patients, mean age 38 years, were reexamined at median 29-year follow-up. 33 patients (55%) had an aSvdH score >0, median score was 4.0 (range 0–313), and 25% of the scores were high (≥53). Most patients with radiographic damage (88%) had both erosions and JSN. 52% of the patients had damage in the wrists, 43% in the MCP joints and 40% in the PIP joints.

The CHR correlated strongly with the aSvdH. Both scores had high correlations with the Juvenile Arthritis Damage Index and the number of joints with limited range of motion (LROM) (r_s_ = -0.688 to 0.743, *p* ≤ 0.001). The aSvdH correlated weakly with measures of disease activity.

The number of joints with LROM, ESR and the HAQ disability score at 15 years and HLAB27 positivity predicted the aSvdH score and the CHR at 29-year follow-up.

**Conclusions:**

The majority of patients with long-term active JIA had modest radiographic damage, but more frequently in wrists than in fingers. The radiographic scores correlated well with measures of disease damage. Restricted mobility in joints at 15 years was the most important predictor of radiographic damage at 29 years.

## Background

Juvenile idiopathic arthritis (JIA) is a chronic inflammatory autoimmune disease which affects the joints of a child. This chronic inflammation can cause growth disturbances, joint space narrowing (JSN) and erosions in the joints. Patients with a polyarticular disease course have the highest probability of attaining joint destructions [1].

Until recently a standard for radiographic assessment in JIA did not exist. Several methods have been suggested and evaluated [2–6]. One has looked for an index joint which could represent the radiographic status of a child with JIA, and found that the joints most often affected in polyarticular disease are the hands and wrists [1, 7–9].

Recently an adapted version of the Sharp/van der Heijde (aSvdH) score has been developed for radiographic scoring of patients with JIA [10]. It has proved to be reliable, has shown good construct validity and is capable of detecting radiographic progression [10]. In most late studies on radiographic outcome in patients with adult rheumatoid arthritis (RA), the method of Sharp and van der Heijde is frequently applied with a focus on hands and feet. We therefore chose to use the aSvdH method to score radiographs of hands/wrists in our adult JIA group.

The Poznanski score, an expression of damage in the carpal area, has been used in several studies of radiological damage of hands/wrists in JIA [5, 6, 11, 12]. The Poznanski score is dependent on a growth plate in the distal radius and thus not suitable for adults. However, the Carpal Height Ratio (CHR) has some similarities with the Poznanski score, assesses carpal damage and is applicable in adults [13, 14].

Nutritional factors which influence mineral homeostasis, differentiation of the osteoblasts and the immune system, such as vitamin D and vitamin A, have not previously been included in long-term studies of radiographic outcome in JIA [15, 16].

There is a lack of long-term radiographic outcome studies of adult patients with JIA. The aim of our study was to describe radiographic damage in hands/wrists of patients with long-term active JIA after 29 years of disease duration, by applying the aSvdH score and the CHR in these patients. We also wanted to assess correlations between radiographic outcome and disease variables after 29 years and evaluate possible predictors of radiographic outcome.

## Methods

### Patients

A total of 260 patients with juvenile idiopathic arthritis (JIA), first time referred to Oslo University Hospital, Rikshospitalet from 1980 to 1985, were reexamined clinically after median 15 years of disease duration and by mailed questionnaires after median 23 years [17, 18]. A total of 176 of these patients were assessed with questionnaires after median 29.6 years [19]. Those who had signs of active disease either at 15, 23 or 29.6 years came to an additional clinical examination. Long-term active disease was defined as having active disease at 15-year follow-up or later. Of the 90 patients examined with long-term active disease, 60 patients (67%) had a history of previous or present arthritis in the wrists and underwent radiographs of hands/wrists.

The patients were classified according to the International League of Associations for Rheumatology (ILAR) criteria for JIA after 15 years of disease duration [18, 20]. Data from baseline was available to fulfil the criteria. Disease onset was defined as the day the physician diagnosed the arthritis.

The study was approved by the Regional Committee for Medical and Health Research Ethics. Written informed consent was obtained from all the participants according to the Declaration of Helsinki.

### Clinical examination

The patients were examined by one of three pediatric rheumatologists at follow-up (BF, VL, AMS). The clinical examination included registration of number of joints with swelling, tenderness and limited range of motion (LROM), number of active joints (swelling or both tenderness and LROM), and physician’s global assessment of disease activity [on a 10 cm Visual Analogue Scale (VAS), where 0 means no disease activity and 10 means very severe disease activity].

We used the Juvenile Arthritis Damage Index (JADI) to assess clinical damage, the Health Assessment Questionnaire (HAQ) and the Juvenile Arthritis Disease Activity Score (JADAS-71) to evaluate physical disability and disease activity and the Medical Outcome Study 36-item Short Form Health Survey (SF-36) to assess quality of life [21–24]. The preliminary criteria for remission in select categories of JIA was applied as described in a previous study on the same cohort [19].

Blood samples were drawn after an overnight fast. Erythrocyte sedimentation rate (ESR), C-reactive protein (CRP), rheumatoid factor (RF) IgM, anti-cyclic citrullinated peptides (anti-CCP) and antinuclear antibody (ANA) were analysed by standard methods. Vitamin A was measured by ultra high-performance liquid chromatography with UV detection and 25-hydroxy vitamin D was measured by liquid chromatography–tandem mass spectrometry.

### Radiographic examinations

Conventional radiographs of the hand/wrist in the posteroanterior view, digital technique, were assessed in consensus by two musculoskeletal radiologists experienced in radiology of children with rheumatic diseases (EK, LT). The aSvdH method was used to score the radiographs of the hands/wrists [10]. The score is based on the assessment of joint space narrowing in 15 areas and bone erosions or deformity in 21 areas, on a 0–4 and a 0–5 point severity scale, respectively. Bone erosions and deformity were considered as equivalent. The total aSvdH score is calculated as the sum of the scores for JSN (range 0–120) and erosion (range 0–210) and ranges from 0 to 330*.* We defined a score above the 75-percentile as a high score/severe damage.

The CHR was calculated as the ratio between the carpal height (measured between the base of metacarpal III and the distal radial articular surface, measured along the axis of metacarpal III) and the length of metacarpal III [13]. The average CHR in normal controls (Belgian volunteers) has been found to be 0.52 ± 0.07 [14].

Six patients had signs of previous operations (prosthesis and/or arthrodesis) in joints scored by the aSvdH method and these joints were given the highest erosion and JSN sores.

Thus we obtained complete aSvdH scores for all the patients. CHR could not be scored in 7 patients due to joint fusion or prosthesis in the third carpometacarpal joint or in the third metacarpophalangeal (MCP) joint.

### Statistical analyses

The differences between 2 patient groups were analyzed by t tests for continuous normality distributed data, Mann–Whitney *U* test for non-normality distributed data and *χ* ^2^ for frequencies. Because of non-normality distribution, we used the Kruskal-Wallis test to analyze differences in the aSvdH scores across the JIA categories. We used a one way analysis of variance with Bonferroni correction to look for differences in CHR between JIA categories. Spearman’s correlation coefficient (r_s_) was used for non-normality distributed variables.

Missing data was observed in up to 7% of all variables except for JADAS-71 (10%) and CHR scores (12%). Missing data was replaced by median substitution except for CHR scores which were replaced by the worst recorded CHR as missing scores were due to bone damage in the wrists which made it impossible to score.

Initial univariate linear regression analyses tested the relation between patient characteristics, HLA alleles, disease variables at baseline, core set variables at 15-year follow-up and aSvdH or CHR scores at 29 years [25]. Subsequent multivariate linear regression analyses were used to identify a set of statistically significant predictors, where variables with a p value <0.05 in the univariate tests were analyzed as possible predictors. Highly intercorrelated independent variables (r >0.7) were avoided. Backward and forward regression models were used.

For all analyses, p values <0.05 (2 tailed tests) were considered statistically significant. All analyses were performed on the SPSS software program (SPSS Inc., Chicago, IL, USA) version 21.0.

## Results

### Patients

**Fig. 1:**
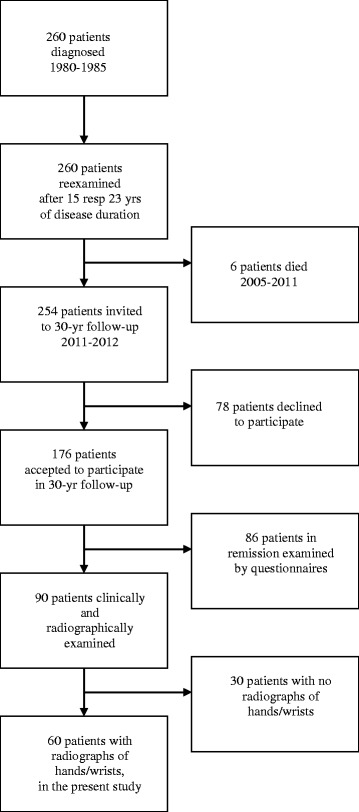
Flow chart of how the 60 patients were selected from the original cohort of 260 patients

Out of the original cohort of 260 patients, 60 patients with long-term active JIA and a history of previous or present arthritis in the wrists underwent radiographs of the hands and wrist joints after median 29.4 years (range 26–38) of disease duration (Fig. 1). We missed one patient, who had swelling in the wrist and two proximal interphalangeal (PIP) joints at 29 years, but underwent no radiographs of the hands, thus not included in the study. The 60 patients had significantly more affected wrists both at 6 months and 15-year follow-up compared to those 200 not examined (*p* ≤ 0.001). Physician’s global, number of active joints, number of joints with LROM, HAQ, SF-36 PCS and patient’s global were all significantly worse at 15-year follow-up in the study group than in the 200 patients not examined (*p* ≤ 0.05, data not shown).

Table 1 shows a description of the patient group. The patients had a median age of 38.2 years (range 30–45) and 70% were female. In general patients had modest disease activity (median 0 swollen joints, 4 joints with LROM, 0 active joints), but 48 patients (80%) had active disease and only 12 patients (20%) were in remission off medication at 29-year follow-up. Four patients (7%) were RF IgM-positive, 6 patients (10%) were anti-CCP-positive and 19 (32%) were ANA positive.

**Table 1:** Demographic and clinical characteristics of 60 patients with JIA after 29 years of disease duration

Characteristics	Value
Age, years	38.2 (30–45)
Female	42 (70)
Disease duration, years	29.4 (26–38)
Number of swollen joints	0 (0–12)
Number of tender joints	1 (0–16)
Number of joints with LROM	4 (0–56)
Number of active joints	0 (0–13)
PGA, VAS cm	1.5 (1–7.6)
Patient’s global well-being, VAS cm	2.0 (1–5.9)
HAQ	0.5 (0–1.6)
JADAS	4.8 (0.2–27.1)
JADI total	6 (0–73)
CRP, mg/l	2.0 (0–38)
ESR, mm/h	6.0 (2–60)
Vitamin A μmol/L	1.6 (0.9–2.4)
Vitamin D nmol/L	64 (26–100)
Patients in remission off medication	12 (20)
Patients with inactive disease on medication	8 (13)
Patients with persistently active disease	40 (67)
Patients on sDMARDs	19 (32)
Patients on biological immunosuppressants	21 (35)
Patients on glucocorticosteroids	6 (10)
Employed^a^	46 (77)

Numbers are median (range) or number (%). ^a^Employed is full or part time

*LROM* limited range of motion, *PGA* physician’s global assessment of disease activity, *JADAS* juvenile arthritis disease activity score, *JADI* juvenile arthritis damage index, *sDMARDs* synthetic disease-modifying anti-rheumatic drugs

### Radiographic scores of the hand and wrist after 29 years

Thirty three patients (55%) had radiographic damage (aSvdH > 0), 9 patients (15%) had aSvdH scores between 1 and 20, 9 patients (15%) had aSvdH scores between 21 and 52, and 15 (25%) had aSvdH scores ≥53 (range 53–313).

**Fig. 2:**
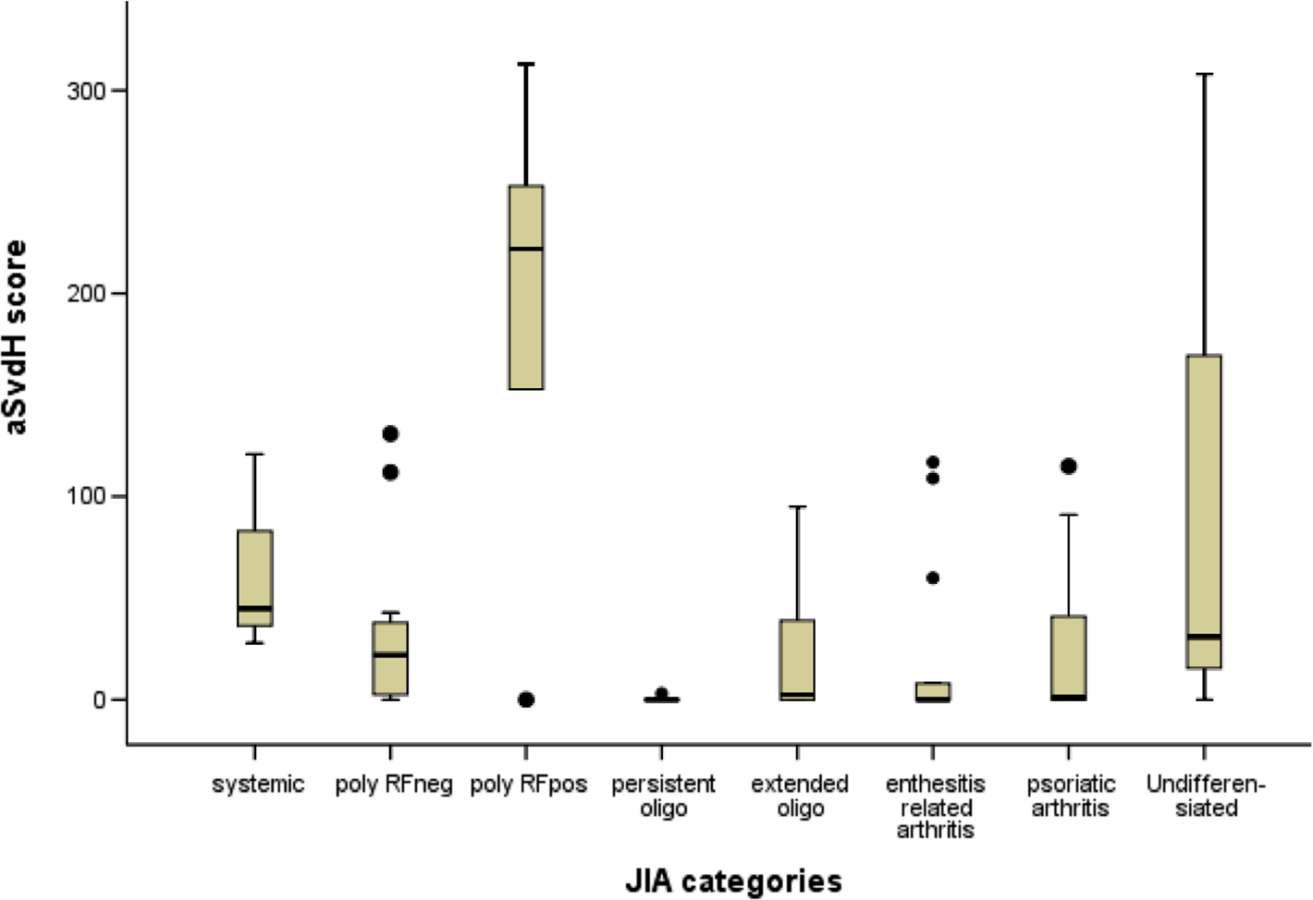
The distribution of the aSvdH scores according to JIA category. aSvdH = adapted Sharp van der Heijde, RF = rheumatoid factor

The aSvdH scores of the radiographs of hands/wrists were median 4 (range 0–313), and the scores of erosions and JSN were 1 (range 0–205) and 3 (range 0–110), respectively (Table 2). Patients with systemic or polyarticular RF-positive JIA had the highest frequency of damage. Patients with polyarticular RF-positive JIA also showed the highest aSvdH scores (median 222, range 0–313) (Table 2 and Fig. 2). Their scores were significantly higher than the scores from patients with polyarticular RF-negative JIA (median 22, *p* = 0.038), extended oligoarticular JIA (median 3, *p* = 0.028), enthesitis related arthritis (ERA) (median 0, *p* = 0.019) and psoriatic arthritis (PsA) (median 1, *p* = 0.028). Likewise, patients with polyarticular RF-positive JIA had significantly worse erosion and JSN scores (median 127 and 95, respectively) than those with polyarticular RF-negative JIA, extended oligoarticular JIA, ERA and PsA (*p* ≤ 0.05). The aSvdH scores were also significantly worse in anti-CCP-positive patients compared to anti-CCP-negative (mean 238 vs 2, *p* = 0.001).

**Table 2:** The aSvdH score in hands and wrists of patients with JIA at 29-year follow-up

JIA categories	No. of patients	No. of patients with a score > 0	Total aSvdH score	Erosion score	JSN score	JADI Articular damage	JADI Extra-articular damage	JADI Total damage
Total	60 (100)	33 (55)	4 (0–313)	1 (0–205)	3 (0–110)	5 (0–68)	1 (0–7)	6 (0–73)
Systemic arthritis	3 (5)	3 (100)	45 (28–121)	25 (10–75)	20 (18–46)	13 (8–24)	1 (0–2)	13 (9–26)
Polyarticular RF-negative	11 (18)	8 (61)	22 (0–131)	5 (0–76)	8 (0–69)	8 (0–36)	1 (0–2)	6 (0–38)
Polyarticular RF-positive	5 (8)	4 (80)	222 (0–313)^a^	127 (0–205)^b^	95 (0–108)^b^	30 (0–68)	1 (0–5)	31 (0–73)
Persistent oligoarticular	5 (8)	1 (20)	0 (0–3)	0 (0–1)	0 (0–2)	0	1 (0–3)	1 (0–3)
Extended oligoarticular	10 (17)	5 (50)	3 (0–95)	0 (0–56)	3 (0–39)	7 (0–24)	1 (0–3)	8 (0–24)
Enthesitis related arthritis	13 (22)	5 (38)	0 (0–117)	0 (0–69)	0 (0–48)	3 (8–28)	1 (0–3)	5 (0–30)
Psoriatic arthritis	10 (17)	5 (50)	1 (0–115)	1 (0–68)	0 (0–47)	3 (0–42)	0 (0–2)	3 (0–42)
Undifferentiated arthritis	3 (5)	2 (67)	31 (0–308)	25 (0–128)	6 (0–110)	7 (2–43)	4 (0–7)	11 (2–50)

Numbers are median (range) or number (%). ^a^Significantly different from scores in patients with polyarticular RF-negative JIA, extended oligoarticular JIA, enthesitis related arthritis and psoriatic arthritis (all *p* < 0.05). ^b^Significantly different from scores in patients with polyarticular RF-negative JIA, persistent oligoarticular JIA, extended oligoarticular JIA, enthesitis related arthritis and psoriatic arthritis (all *p* < 0.05). *aSvdH* adapted Sharp van der Heijde, *JSN* joint space narrowing, *JADI* juvenile arthritis damage index

We found that 2 patients (3%) had only erosions, 2 patients (3%) had only JSN, 29 patients (48%) had both erosions and JSN (scores > 0) and 27 patients (45%) had no radiographic damage. The total erosion score correlated with the total JSN score (r_s_ = 0.945, *p* < 0.001) and the erosion scores in right and left hand correlated with the respective JSN scores (r_s_ = 0.950 and r_s_ = 0.897, respectively, *p* < 0.001).

When dividing radiographic damage into carpal area, MCP and PIP joints, we found that 31 patients (52%) had damage in the carpal area, 26 (43%) had in MCP joints and 24 (40%) had damage in PIP joints (*p* < 0.001).

The mean CHR was 0.49 ± 0.08 (Table 3). The patients with polyarticular RF-positive JIA had significantly lower CHR than patients with polyarticular RF-negative JIA, persistent oligoarticular JIA, extended oligoarticular JIA, ERA and PsA (*p* ≤ 0.005 for all). Patients with systemic JIA had significantly lower CHR than patients with persistent oligoarticular JIA, ERA and PsA (*p* < 0.05). Eight patients had a mean score ≤0.38. The mean CHR correlated significantly with aSvdH scores (r_s_ = -0,779 *p* < 0.001).

**Table 3:** The CHR of patients with JIA at 29-year follow-up

JIA categories	No. of patients	CHR Right	CHR Left	Mean CHR
Total	60 (100)	0.52 (0.31–.64)	0.52 (0.31–0.63)	0.49 (0.08)
Systemic arthritis	3 (5)	0.32 (0.31–0.48)	0.34 (0.31–0.48)	0.37 (0.09)^a^
Polyarticular RF-negative	11 (18)	0.51 (0.43–0.57)	0.51 (0.31–0.59)	0.50 (0.05)
Polyarticular RF-positive	5 (8)	0.31 (0.31–0.53)	0.31 (0.31–0.53)	0.35 (0.10)^b^
Persistent oligoarticular	5 (8)	0.56 (0.52–0.58)	0.56 (0.52–0.56)	0.55 (0.02)
Extended oligoarticular	10 (17)	0.50 (0.31–0.55)	0.51 (0.47–0.56)	0.50 (0.04)
Enthesitis related arthritis	13 (22)	0.55 (0.42–0.64)	0.54 (0.31–0.62)	0.54 (0.06)
Psoriatic arthritis	10 (17)	0.52 (0.32–0.62)	0.52 (0.45–0.63)	0.52 (0.06)
Undifferentiated arthritis	3 (5)	0.40 (0.31–0.53)	0.40 (0.31–0.58)	0.42 (0.12)

Numbers are median (range), number (%) or mean (SD). ^a^Significantly different from scores in patients with persistent oligoarticular arthritis, enthesitis related arthritis and psoriatic arthritis (*p* < 0.05). ^b^Significantly different from scores in patients with polyarticular RF-negative JIA, persistent oligoarticular JIA, extended oligoarticular JIA, enthesitis related arthritis and psoriatic arthritis (*p* ≤ 0.005). *CHR* carpal height ratio

### The relation between radiographic scores and measures of disease activity and damage

After 29 years, the total aSvdH, erosion and JSN scores correlated strongly with JADI scores and the number of joints with LROM (r_s_ = 0.659 to 0.721, *p* < 0.001, Table 4). The CHR had also a high correlation with JADI scores and joints with LROM (r_s_ = -0.660 to-0.645, *p* < 0.001). The aSvdH score had weak correlations with JADAS-71 and CRP (r_s_ = 0.266 to 0.268, *p* < 0.05). The erosion and JSN scores correlated with HAQ (r_s_ = 0.278 and r_s_ = 0.259, respectively, *p* < 0.05). All the radiographic scores of the hands/wrists had a weak to moderate correlation with vitamin A levels (r_s_ = -0.299 to 0.430, *p* < 0.05). Vitamin D levels did not correlate consistently with any of the scores, nor did the number of swollen, tender or active joins, pain, ESR, well-being or variables on smoking habits.

**Table 4:** Correlations between radiographic scores and measures of disease damage and disease activity at 29-year follow-up

	aSvdH scores	Erosion scores	JSN scores	Mean CHR
Variables	4.0 (0–313)	1.0 (0–205)	3.0 (0–110)	0.52 (0.31–0.63)
JADI total	0.706^a^	0.721^a^	0.714^a^	−0.660^a^
Joints with LROM	0.669^a^	0.659^a^	0.665^a^	−0.645^a^
JADAS	0.268^b^	0.255^b^	0.266^b^	NS
HAQ	NS	0.278^b^	0.259^b^	NS
CRP	0.266^b^	NS	0.273^b^	NS
Vitamin A	−0.272^b^	−0.275^b^	−0.299^b^	0.430^a^

Numbers are median scores (range) and Spearman’s correlation coefficients. The radiographic scores did not correlate with the number of swollen, tender and active joints, pain, well-being, ESR or vitamin D. ^a^ *p* ≤ 0.001, ^b^ *p* < 0.05. *aSvdH* adapted Sharp van der Heijde, *JSN* joint space narrowing, *CHR* carpal height ratio, *JADI* juvenile arthritis damage index, *LROM* limited range of motion, *JADAS* juvenile arthritis disease activity score, *NS* not significant

The aSvdH score for those in remission at 29 years was significantly lower than the score for those with active disease [median 0 (range 0–45) vs median 11.5 (range 0–313), *p* = 0.038]. When analysing JSN scores separately, there was a significantly lower JSN score in those in remission compared to those with active disease [median 0 (range 0–20) vs median 6 (range 0–110), *p* = 0.023]. We found the same tendency for erosions, but the difference was not statistically significant (*p* = 0.054).

At 15-year follow-up, 53 (88%) of the 60 patients had used median 2.5 (range 1–7) different synthetic(s) DMARDs during their disease course (data not shown). At 29 years, 33 (69%) out of total 48 patients with active disease, used sDMARDs, biological immunosuppressants and/or prednisolone. When comparing these patients with those not treated with sDMARDs, biological immunosuppressants and/or prednisolone (*n* = 15), those on treatment had significantly higher aSvdH scores [median 28 (range 0–313) vs median 0 (range 0–113), *p* = 0.014], but there were no significant differences in disease activity measures (ESR, CRP, number of active joints, joints with LROM, patient’s and physician’s global assessment). There were no significant differences between aSvdH score or CHR in those employed versus those not employed (data not shown).

### Predictors of radiographic scores from clinical variables at baseline and 15 years

All the core set variables from 15-year follow-up (except patient’s global assessment of well-being), HLA-DR4, HLA-B27, onset and course type were associated with the aSvdH scores and CHR after 29 years (Table 5). In a multivariate linear regression analysis a higher number of joints with LROM and ESR from 15-year follow-up, predicted a higher aSvdH score at 29-year follow-up, R^2^ = 65%. The number of joints with LROM and HAQ at 15 years and HLA-B27 positivity were predictors of the mean CHR at 29 years, R^2^ = 51%.

**Table 5:** Predictors of the aSvdH score and mean CHR at 29-year follow-up

	aSvdH				Mean CHR			
	Univariate analyses		Multivariate analyses		Univariate analyses		Multivariate analyses	
Variables assessed within 15 years:	B (95% CI)	*P* value	B (95% CI)	*P* value	B (95% CI)	*P* value	B (95% CI)	*P* value
Oligoarticular onset^a^	−48.1 (−85.3 to −10.9)	0.012			0.047 (0.005 to 0.090)	0.028		
Polyarticular course^b^	NA	NA			−0.060 (−0.120 to 0.000)	0.048		
HLA-DRB1*04 positive	67.2 (28.8 to 105.5)	0.001			NA	NA		
HLA-B27 positive	NA	NA			0.057 (0.011 to 0.102)	0.016	0.047 (0.013 to 0.082)	0.009
No. joints with LROM at 15 years^c^	5.9 (4.7 to 7.2)	<0.001	4.8 (3.4 to 6.3)	<0.001	−0.005 (−0.007 to−0.004)	<0.001	−0.004 (−0.006 to−0.002)	<0.001
ESR at 15 years, mm/h	2.6 (1.7 to 3.5)	<0.001	1.1 (0.3 to 1.9)	0.010	−0.002 (−0.003 to−0.001)	<0.001		
CRP at 15 years, mg/l	2.3 (1.2 to 3.3)	<0.001			NA	NA		
HAQ at 15 years	53.5 (22.2 to 84.7)	0.001			−0.065 (−0.100 to −0.031)	<0.001	−0.042 (−0.071 to −0.012)	0.007
Total time with active disease at 15-year follow-up, years	5.1 (1.0 to 9.1)	0.015			−0.007 (−0.011 to −0.002)	0.005		

^a^Oligoarticular onset: ≤ 4 active joints first 6 months, from all JIA categories except polyarticular JIA. ^b^Polyarticular course: having in total ≥ 5 active joints after 6 months of disease, from all JIA categories except persistent oligoarticular JIA. ^c^Number of joints with LROM at 15 years correlated highly (r >0.7) with physician’s global assessment and number of active joints at 15 years so only joints with LROM was chosen in the model. *aSvdH* adapted Sharp van der Heijde, *CHR* carpal height ratio, *LROM* limited range of motion, *NA* not applicable

## Discussion

In the present study of 60 patients with long-term active JIA and clinical arthritis in the wrist during their disease course, more than half of these patients had radiographic damage measured by the aSvdH score in the hands/wrists after 29 years. The damage was modest in most patients, but 25% had severe damage. Patients with polyarticular RF-positive or anti-CCP-positive JIA had the worst damage. The majority of patients with radiographic damage had both erosions and JSN. Most damage occurred in the carpus area compared to the rest of the hand. Both the radiographic scores correlated strongly with other measures of disease damage at 29-year follow-up. The number of joints with LROM at 15 years was the most important predictor of the radiographic scores after 29 years of disease duration.

We found that 55% of the patients with long-term active disease and previous or present arthritis in the wrist had developed radiographic damage in the hands/wrists. There are few studies on radiographic long-term outcome in JIA patients, and reports of radiographic outcome especially in hands/wrists are even more scarce. A study from the sixties reported presence of erosions in hands/wrists of 68% of patients with juvenile rheumatoid arthritis (JRA) after 18 years [26]. The majority of their patients had polyarticular disease and 28% of them were RF-positive. Damage in this study refers to erosions only which is not comparable to the aSvdH score. Another study reported radiographic damage in hands/wrists in 79% of patients with polyarticular JIA (54% RF-positive) after 13.3 years compared to 86% in patients with RA (79% RF-positive, not significant) after 11 years [27]. Other studies have reported worse radiographic outcome in patients with RA compared to patients with JIA [28].

Patients with polyarticular RF-positive JIA showed the most serious damage in hands/wrists and most of them had been through surgical operations in the wrist. It is well known that patients in this JIA category have the most aggressive disease and thus the most damage [19, 29–32]. Studies have shown that after 10–15 years of disease duration the most severely affected joints in RF-positive polyarticular juvenile arthritis were hands/wrists, and erosions were most commonly seen in these joints [1, 8, 27, 33]. We found that aSvdH scores were significantly worse in anti-CCP-positive patients compared to anti-CCP-negative patients, which is in harmony with other studies [34, 35].

In the present study most patients (29/33) with radiographic damage had both erosions and JSN after 29 years and very few had only erosions or JSN. Erosion and JSN scores correlated highly. There are few studies that have assessed this issue up to now in JIA [36]. One study found that erosions and JSN occurred at almost equal frequencies in patients with polyarticular JRA examined after 8 years of disease [32].

Patients had more often radiographic damage in the carpal area than in MCPs and PIPs in our study. A recent study supports this as they reported more damage in carpus and less in MCPs after up to 10 years of disease duration in patients with JIA and polyarticular course [36]. On the other hand, another study found damage in the MCP joints of all patients with polyarticular JIA after 13 years of disease duration and in 97% of the carpal area [27].

We chose to include the CHR to compare radiographic damage in the carpal area as measured by aSvdH and CHR. Both methods defined patients with polyarticular RF-positive JIA as having the worst damage and patients with systemic JIA as having the second worst damage. A study which applied the Poznanski method also found significant damage in the wrist of these two JIA categories [37]. The aSvdH scores correlated strongly with the CHR, in the same way as other studies have shown moderate to high correlations between the Sharp/aSvdH and the Poznanski score [6, 10]. Thus the aSvdH score performs well in scoring wrist damage in JIA.

We observed high correlations between the radiographic scores aSvdH or CHR and scores for disease damage (JADI and joints with LROM), and lower correlations for the disease activity score (JADAS-71). This is in harmony with other studies on JIA which have reported associations between radiographic measures and JADI and with the number of joints with LROM [6, 10, 11, 21, 32, 38]. Also in adult RA radiographic scores are reported to be associated with other measures of disease damage and disability [39, 40].

Eighty percent of our patients had active disease, but only 69% of them used sDMARDs, biological immunosuppressants and/or prednisolone. This may indicate that some of them are not optimally treated, but on the other hand they may have a mild disease course as the aSvdH scores of those not on advanced treatment were in the lower range.

There were persistent correlations between vitamin A levels and all the radiographic scores. Fifteen percent of the patients had vitamin A levels ≤ 1.2 μmol/L and this subgroup had the highest radiographic scores. Retinoic acid is known to be involved in proliferation and differentiation of osteoblasts and low levels of retinoic acid can have a harmful effect on bone metabolism [41, 42]. However, this correlation needs to be confirmed. There is a lot of research in the field of retinoic acid and its importance in regulation of T cells and other immune cells [43, 44] . Alternatively, our finding of an association with low vitamin A status may be connected to deficiency in immune cells and thus the inflammatory process and not directly on bone metabolism.

We found that the number of joints with LROM and ESR from 15-year follow-up were predictors of aSvdH after 29 years. This is supported by previous studies, which have found ESR as an important predictor for joint destruction [45, 46]. We have in an earlier 3-year follow-up study, revealed the number of joints with LROM as a predictor of radiographic changes [7]. Others have reported that continuing disease activity is a predictor of joint damage [47]. HLA-B27 positivity protected against radiographic damage measured as CHR. This seems to be explained by the fact that 11 out of 18 HLAB27 positive patients had ERA and they had less damage in the wrists as shown in Tables 2 and 3. Alternatively HLA-B27 represents a marker for ERA. When ERA patients were excluded from the analysis, HLA-B27 was no longer a predictor of CHR. On the other hand, a previous study found HLA-B27 positivity associated with worse radiographic outcome in JIA [9].

The strength of the study is the long-term follow-up over 29 years of this cohort that has been described in several publications [17–19, 46, 48]. There are however some limitations of the study. We have examined only patients with long-term active disease who also had current or previous clinical arthritis in the wrist joints, thus a selection of those with severe disease. Optimally we could have taken radiographs of all the patients, including those who went into remission before 15 years. We would have registered the progression of bone damage compared to previous radiographs, but these were unfortunately not available. There were few patients with persistent oligoarticular JIA, therefore their results must be interpreted with caution. The radiographic method we used, the aSvdH score, is evaluated for children with JIA, but we have used it in adults with JIA. However, the Sharp van der Heijde method is widely used in adults with RA and the main difference in this adapted version is the adding of locations in the carpal area in which patients with JIA have more changes than adults with RA [10]. Another limitation is that the JADAS is not validated for adults with JIA. On the other hand, we used the JADAS in a recent study and found that the JADAS correlated strongly with categories of disease activity in adult patients, but JADAS may capture some other aspects of the burden of disease like damage and overall well-being in adults [19].

We did not perform intra- and interrater reliability testing among raters of the radiographs. Our two raters agreed by consensus. The Sharp/Sharp van der Heijde method has previously been evaluated as having good reliability and reproducibility [6, 49], and a good inter- and intraobserver agreement has been demonstrated for the aSvdH in JIA [10].

## Conclusions

In the present cohort of patients with prolonged active JIA and wrist involvement during 29 years of disease duration, we found that about half of the patients had radiographic damage in hands/wrists. Changes were modest for most patients, but one out of four had severe damage. Patients with JIA and arthritis in hands/wrist should be examined regularly with radiographs and especially patients who have joints with LROM. Our patients were diagnosed early in the methotrexate era. Patients nowadays receive more aggressive therapy and will most likely have better radiographic outcome in long-term follow-up than patients in the present study. Similar long-term radiographic outcome studies are needed to continually survey the effectiveness of the present medical regimes, and hopefully document improved outcomes compared to the present study in the future.

## Abbreviations

ANA: Antinuclear antibody
anti-CCP: Anti-cyclic citrullinated peptides
aSvdH score: The adapted version of the Sharp van der Heijde score
CHR: Carpal height ratio
CRP: C-reactive protein
ERA: Enthesitis related arthritis
ESR: Erythrocyte sedimentation rate
HAQ: Health assessment questionnaire
HLA: Human leukocyte antigen
ILAR: International League of Associations for Rheumatology
JADAS: Juvenile arthritis disease activity score
JADI: Juvenile arthritis damage index
JIA: Juvenile idiopathic arthritis
JSN: Joint space narrowing
LROM: Limited range of motion
MCP: Metacarpophalangeal
PIP: Proximal interphalangeal
PsA: Psoriatic arthritis
RA: Rheumatoid arthritis
RF: Rheumatoid factor
sDMARDs: Synthetic disease-modifying anti-rheumatic drugs
SF-36: Medical outcome study 36-item short form health survey
VAS: Visual analogue scale
